# Retraction: Urinary Bladder Hernia: A Rare Cause of Urinary Frequency

**DOI:** 10.7759/cureus.r119

**Published:** 2024-01-25

**Authors:** Abdurahman A Aldubaikhi, Sultan A Albeabe, Ahlam M Alfaraj, Talal M Al Thobaiti, Salman S Shaaban, Thamer S Aloqaili, Alhanouf S Aldossary, Sami N Alanazi, Ziyad F Althobaiti, Ebtehal Z Alqahtani, Abdulrahman Z Alqahtani, Ali S Alsoghayer, Abdulhalim A Alabdullatif, Abdulwahab A Altammar, Faisal Al-Hawaj

**Affiliations:** 1 College of Medicine, Qassim University, Qassim, SAU; 2 College of Medicine, Arabian Gulf University, Manama, BHR; 3 College of Medicine, Jordan University of Science and Technology, Irbid, JOR; 4 College of Medicine, King Abdulaziz University, Jeddah, SAU; 5 College of Medicine, University of Dammam, Dammam, SAU; 6 College of Medicine, AlMaarefa University, Ad Diriyah, SAU; 7 College of Medicine, Northern Border University, Arar, SAU; 8 College of Medicine, Taif University, Taif, SAU; 9 College of Medicine, Jazan University, Jazan, SAU; 10 College of Pharmacy, Jazan University, Jazan, SAU; 11 College of Medicine, University of Malta, Msida, MLT; 12 College of Medicine, Imam Abdulrahman Bin Faisal University, Dammam, SAU

The Editors-in-Chief have retracted this article. Concerns were raised regarding the identity of the authors on this article. Specifically, Faisal Alhaway and Malak Shammari have stated that they were added to this article without their knowledge or approval. The identity of the other authors could also not be verified. As the appropriate authorship of this work cannot be established, the Editors-in-Chief no longer have confidence in the results and conclusions of this article.

